# Oral function tests in spinal muscular atrophy: closing the diagnostic gap in severely affected adult patients: A prospective observational study

**DOI:** 10.1007/s00056-025-00597-8

**Published:** 2025-06-24

**Authors:** Teresa Kruse, Sara Portegys, Diana Leflerovà, Annette Cap, Brunhilde Wirth, Raoul Heller, Svenja Neuhoff, Tim Hagenacker, Bert Braumann, Gilbert Wunderlich

**Affiliations:** 1 Department of Orthodontics, Faculty of Medicine and University Hospital Cologne, University of Cologne, Kerpener Str. 32, 50931 Cologne, Germany; 2 Center for Rare Diseases Cologne, University of Cologne, Kerpener Str. 62, 50937 Cologne, Germany; 3 Institute of Human Genetics, University of Cologne, Kerpener Str. 34, 50931 Cologne, Germany; 4 Center for Molecular Genetics, University of Cologne, Kerpener Str. 34, 50931 Cologne, Germany; 5 Genetic Health Service NZ—Northern Hub, Auckland District Health Board, Auckland City Hospital, Grafton Rd, Grafton, 90–102 NZ-Auckland 1010, New Zealand; 6 Department of Neurology and Center for Translational Neuro- and Behavioral Sciences (C-TNBS), University Hospital Essen, Hufelandstr. 55, 45147 Essen, Germany; 7 Department of Neurology, Faculty of Medicine and University Hospital Cologne, University of Cologne, Kerpener Str. 62, 50937 Cologne, Germany

**Keywords:** Bulbar neuromuscular function, Ceiling effect, Bite force, Tongue pressure, Malocclusion, Bulbäre neuromuskuläre Funktion, Ceiling-Effekt, Kaukraft, Zungendruck, Malokklusion

## Abstract

**Purpose:**

In advanced stages of spinal muscular atrophy (SMA), established motor scores are unable to distinguish between the different degrees of remaining motor function. Bulbar muscles are affected at a later stage. The aim of the present study was to test whether oral function tests are able to better discriminate motor function than established scores and to replicate known associations between disease-related altered craniofacial anatomy and oral dysfunction in SMA.

**Methods:**

A total of 43 adult individuals with SMA (mean age 39.7 ± 12; 25 men, 18 women) were included in this prospective, cross-sectional study. Oral function was measured using a piezoelectric sensor system and an Iowa Oral Performance Instrument (IOPI) device. Data from oral function tests and established motor scores were analyzed with regard to a possible floor or ceiling effect. It was tested to what extent SMA patients with different malocclusions presented with variable scores.

**Results:**

Patients differed in ambulatory and treatment status (15 ambulatory vs. 28 nonambulatory; 35 treated vs. 8 nontreated) and orthodontic findings (22 with a class II molar relationship and increased overjet, 35 with posterior crossbite). In contrast to the oral function tests, some of the established motor scores showed a clear floor effect. Statistically significant associations were identified between reduced oral function values and an enlarged overjet, a class II molar relationship, and a posterior crossbite. This should be taken into account in neuromuscular evaluations.

**Conclusion:**

In severely affected patients, oral function tests appear to be superior to established motor scores and fill a diagnostic gap in research and clinical practice.

## Abstract (de)

**Ziel:**

Im fortgeschrittenen Stadium der spinalen Muskelatrophie (SMA) sind etablierte motorische Scores nicht in der Lage, zwischen verschiedenen Schweregraden der verbleibenden motorischen Funktion zu unterscheiden. Die bulbären Muskeln sind vergleichsweise spät betroffen. Ziel der vorliegenden Studie war es, zu prüfen, 1), ob orale Funktionstests die motorische Funktion besser unterscheiden können als etablierte Scores und 2), ob sich bekannte Assoziationen zwischen krankheitsbedingt veränderter kraniofazialer Anatomie und oraler Dysfunktion bei SMA replizieren lassen.

**Methode:**

Insgesamt nahmen 43 erwachsene Personen mit SMA (Durchschnittsalter: 39,7 ± 12 Jahre, 25 Männer, 18 Frauen) an der prospektiven Querschnittsstudie teil. Ihre orale Funktion wurde mithilfe eines piezoelektrischen Sensorsystems und eines IOPI (Iowa Oral Performance Instrument)-Geräts gemessen. Die Daten aus den oralen Funktionstests und den etablierten motorischen Scores wurden hinsichtlich möglicher Floor- bzw. Ceiling-Effekte analysiert. Zudem wurde geprüft, inwieweit SMA-Patient:innen mit unterschiedlichen Malokklusionen in den Testergebnissen variierten.

**Ergebnisse:**

Die Patient:innen unterschieden sich hinsichtlich ihrer Gehfähigkeit und ihres Behandlungsstatus’ (15 gehfähige vs. 28 nicht gehfähige; 35 behandelte vs. 8 nicht behandelte) sowie ihrer kieferorthopädischen Befunde (22 mit einer Klasse-II-Molarenrelation und vergrößertem Overjet, 35 mit posteriorem Kreuzbiss). Im Gegensatz zu den oralen Funktionstests zeigten einige der etablierten motorischen Scores einen deutlichen Floor-Effekt. Statistisch signifikante Zusammenhänge wurden zwischen reduzierten oralen Funktionswerten und einem vergrößerten Overjet, einer Klasse-II-Molarenrelation sowie einem posterioren Kreuzbiss festgestellt. Dies sollte bei jeder neuromuskulären Beurteilung der oralen Funktion berücksichtigt werden.

**Schlussfolgerung:**

Bei schwer betroffenen Patient:innen scheinen orale Funktionstests etablierten motorischen Scores überlegen zu sein. Sie schließen so eine diagnostische Lücke in Forschung und Praxis.

## Introduction

Spinal muscular atrophy (SMA) is a progressive neuromuscular disorder characterized by degeneration of motor neurons in the spinal cord. Due to the low incidence of 1 in 7000 live births, it is considered a rare disease [1, 2]. SMA is caused by biallelic deletions and/or point mutations of the survival of motor neuron 1 (*SMN1*) gene [3, 4]. The survival motor neuron 2 (*SMN2*) gene is a homologous copy of *SMN1*. However, only 10% of the SMN protein produced from this gene is fully functional. The more SMN2 copies, the more full-length SMN protein is produced [3, 5].

Historically, four different types of SMA have been defined. Infants with type 1 SMA are very weak and unable to sit unsupported. Type 2 patients are not ambulatory but are able to sit independently. Ambulatory patients with childhood onset are classified as type 3, and ambulatory patients with adult onset are classified as type 4 [6].

As SMA progresses, oral function becomes increasingly impaired, including difficulties in chewing, swallowing, and limited mouth opening [7, 8]. Impaired oral function can lead to weight loss, which further worsens the general condition [9]. Restricted mouth opening can lead not only to poor oral hygiene but also to life-threatening complications during intubation [9]. The risk of severe choking due to inadequate mastication and the risk of aspiration pneumonia as a result of dysphagia increase [10, 11].

Patients with SMA show an altered craniofacial anatomy. The impaired neuromuscular function negatively influences the skeletal development of the growing individual. Typical dysgnathia in patients with neuromuscular diseases including SMA are a skeletal class II with an enlarged overjet, a skeletal anterior open bite and a narrow, high palate with a posterior crossbite [12–14]. These dysgnathia are known to be associated with reduced masticatory strength [15–17]. When evaluating oral function in SMA, the relationship between form and function should be considered, as the anatomical conditions may result in reduced capabilities that are not directly related to the actual limitation in neuromuscular function. In long-term evaluations, such as monitoring treatment effects, anatomical limitations may persist even if neuromuscular function has improved.

Nusinersen (Biogen, Cambridge, MA, USA) is an antisense oligonucleotide correcting the splicing of SMN2 pre-mRNA and, thus, increasing the production of the complete SMN protein. Its therapeutic benefit is uncontroversial in newborns, children and adolescents [18, 19]. The impact on residual motor function in adult patients at an advanced stage of SMA is less clear [20], likely due to established motor scores being either inapplicable to this subgroup or insufficiently sensitive to detect marginal changes. As severely affected individuals score at the lower end of these scales, a floor effect is inevitable. In a similar vein, difficulties in differentiating less affected SMA patients also exist at the upper end of the scale (i.e., ceiling effect). As with all ceiling or floor effects, they make it difficult to discriminate between subjects at either extreme of the spectrum [21, 22], increase bias and uncertainty, and make the affected measure insensitive to change [23, 24].

The Hammersmith Functional Motor Scale Expanded (HFMSE) and the Revised Upper Limb Module (RULM) are the most widely used motor scores for patients with late-onset SMA. They are often complemented by the 6‑Minute Walk Test (6 MWT) and the Revised Amyotrophic Lateral Sclerosis Functional Rating Scale (ALSFRS-R), which includes some questions on bulbar and oral function [25]. Measures specifically targeting oral function, such as the Bogenhausen Dysphagia Score (BODS), are seldom used [26, 27]. Despite their wide acceptance, HFMSE and RULM are at risk of missing possible changes at the extreme ends of the spectrum of physical abilities [28]. Similarly, in patients without upper limb weakness, the RULM also suffers from a ceiling effect [29]. The 6 MWT helps to identify fatigability-related changes in ambulatory SMA patients and addresses the diagnostic gap at the higher end of motor abilities [30]. However, it is only applicable to ambulatory patients and cannot address diagnostic needs at the lower end of the scale. Some authors have suggested that the floor effect, which significantly limits the test’s interpretability, make the HFMSE unsuitable for long-term follow-up in adult patients with SMA types 1, 2 and 3 [28]. For the RULM, a mild floor effect has also been observed in nonsitters [20, 31]. The need for clinical tools that can assess patients with SMA who have severely impaired functional abilities remains urgent [32].

Other methodological approaches such as electrophysiological and video fluoroscopic examinations, questionnaires, and magnetic resonance imaging (MRI) scans have been able to confirm reduced oral function in SMA patients [8, 33], but have not become routine. Measurement of maximum bite force as a quantitative method was used early on in SMA patients and showed reduced force levels of 19–50% compared to healthy controls [33, 34]. Bruggen et al. showed that mandibular dysfunction, including reduced active mouth opening in SMA types 2 and 3, correlated with bulbar involvement [33]. Not only maximum bite force, but also reduced tongue strength is associated with impaired bulbar and upper limb function in SMA, suggesting that they are useful biomarkers not only in SMA [35], but also in other neuromuscular disorders [36–38]. A combination of different oral function tests has been found to best capture the neuromuscular limitations in SMA. However, there are only a few studies using such a multimodal approach [39, 40]. The association between posterior crossbite, open bites and reduced muscle strength has been described in other muscular dystrophies, as well as in people without neuropathy but with weak muscles [12, 13, 38]. It is therefore reasonable to also consider orthodontic findings in a multimodal approach.

The aim of this study was to further evaluate the diagnostic potential of oral function tests on two levels. First, it aimed to show how oral function tests can fill the diagnostic gap in severely affected SMA patients where established motor scores are limited by floor effects. Second, it was intended to provide preliminary data on oral function in SMA patients, taking into account their particular craniofacial characteristics. The objective was to expand the anatomical–orthodontic knowledge of the influence of malocclusion on oral function in SMA, with a view to account for possible biases in the neuromuscular evaluation of these patients.

## Methods

### Study design and study period

This observational, prospective, cross-sectional multicenter study was conducted at the Departments of Neurology at the University Hospitals of Cologne and Essen, Germany and approved by the Ethics Committees of the Medical Faculties of the two sites (Reference Number 19-1137; Reference Number 21-9851-BO). Recruitment commenced in July 2020, with the first measurements taken until the end of the cross-sectional study period in December 2022. The sample size was determined by the number of eligible patients who were willing to participate. The study design complied with the strengthening the reporting of observational studies in epidemiology (STROBE) guidelines. The trial has been preregistered with the German Clinical Trials Register (DRKS) under trial ID DRKS00015842.

### Subjects

The sample initially consisted of 44 adult patients with genetically confirmed 5q-SMA. The inclusion criteria were defined as follows: genetically confirmed 5q-associated SMA, age ≥ 18 years, mouth opening of at least 10 mm, sufficient dentition in the posterior region, and available data on established motor scores (HFMSE, RULM, ALSFRS‑R, and 6 MWT). Subjects were excluded from the study if they exhibited no remaining masticatory function (or no posterior teeth), required permanent ventilatory support, or had an extremely restricted mouth opening (less than 10 mm) that precluded the positioning of a sensor. Applying these exclusion criteria, 1 patient had to be excluded due to a mouth opening of only 8 mm. Accordingly, 43 individuals were included. This sample has already been used to check oral function data for the validity of the measurements [39]. All patients provided informed consent.

The sample consisted of 25 males and 18 females (Table 1). Six patients were Asian (14.3%), 36 patients were Caucasian (85.7%) and for 1 patient there was no information on ethnicity. Patients were diagnosed with SMA type 2 (12 patients, 27.9%) or 3 (31 patients, 72.1%). The mean age of the patients at first examination was 39.7 ± 12.0 years (median 37) ranging between 20 years and 65 years (patients with type 2: 33.8 ± 8.3 and with type 3: 42.0 ± 12.5 years). None of the patients received causal treatment in childhood. At the time of data collection, 35 patients had received at least the first three doses of nusinersen according to the routine clinical practice and 8 patients had not received any treatment. Motor scores were performed in 28 nonambulatory and 15 ambulatory patients. In one case, a single motor score (HFMSE) was not performed.

**Table 1:** Sample characteristics Charakteristika der Stichprobe

		SMA type 2		SMA type 3		Overall	
		*N* = 12 (27.9%)		*N* = 31 (72.1%)		*N* = 43 (100%)	
		Mean ± SD	Min–Max	Mean ± SD	Min–Max	Mean ± SD	Min–Max
Age		33.8 ± 8.3	20–47	42 ± 12.5	26–65	39.7 ± 12	20–65
Gender	Male	–	6 (50%)	–	19 (61.3%)		25 (58.1%)
	Female		6 (50%)		12 (38.7%)		18 (41.9%)
Ethnicity	Caucasian Asian		11 (91.7%)		25 (83.3%)		36 (85.7%)
			1 (8.3%)		5 (16.7%)		6 (14.3%)
Ambulatory	Yes	–	0 (0%)	–	15 (48.4%)		15 (34.9%)
	No		12 (100%)		16 (51.6%)		28 (65.1%)
Nusinersen therapy	Yes		9 (75%)		26 (83.9%)		35 (81.4%)
	No		3 (25%)		5 (16.1%)		8 (18.6%)

*SD* standard deviation; *N* sample size; *Min* minimum; *Max* maximum

### Oral function tests

For the oral function tests, each SMA patient was visited twice with an interval of about 1 week. No nusinersen application or other medical intervention took place within these days to not interfere with the administration of nusinersen (lumbar puncture) immediately before or between the two oral function tests. The tests were conducted prospectively at two sites. Two orthodontists and one dentist performed the measurements alternately after standardized administration procedures. The measurement protocol followed recommendations from the literature that have proven successful in both healthy and SMA patients [33, 34, 40, 41]. Calibration of the examiners took place before the first measurement. Interrater reliability was tested in advance on healthy subjects and showed at least a ‘substantial agreement’ for tongue pressure endurance (intraclass correlation coefficient [ICC] 0.67) up to an ‘excellent agreement’ for bite force endurance (ICC 0.95). Intrarater reliability was “excellent” for all oral function tests [39]. Each bite force sensor for each subject was equilibrated prior to the first measurement. A postmeasurement calibration process, in which a defined force was applied to the sensor via the participants’ plaster models, minimized sensor-specific measurement inaccuracies and provided the basis for measuring absolute bite force values in newtons. The calibration of the Iowa Oral Performance Instrument (IOPI) for measuring tongue pressure was checked regularly as recommended in the IOPI manual.

**Fig. 1:**
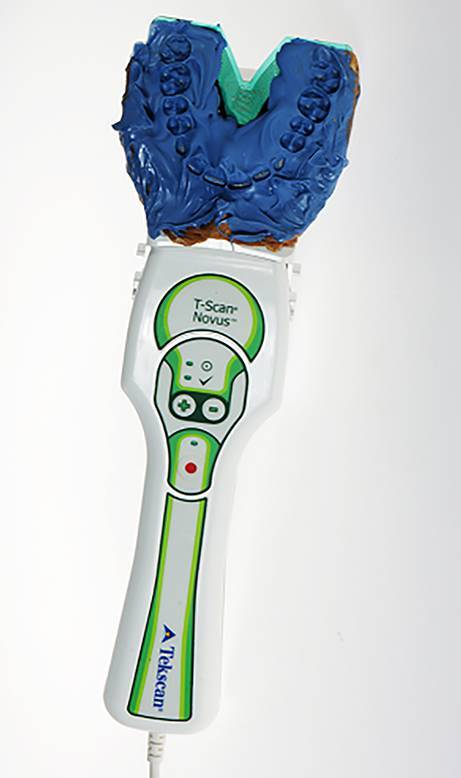
Adapted piezoelectric T‑scan sensor foil with individualised surface for measuring maximum bite force and bite force endurance over the entire dental arch Adaptierte piezoelektrische T‑Scan-Sensorfolie mit individualisierter Oberfläche zur Messung der maximalen Kaukraft und der Kaukraftausdauer über den gesamten Zahnbogen

To measure maximum bite force and bite force endurance, a piezoelectric sensor system (T-Scan handle with a T-Scan sensor combined with the I‑Scan software; Tekscan, Inc., South Boston, MA, USA) was used. The surface of the sensor was adapted chairside to the patient’s dental situation using dental silicone (Flexitime Automix Light Flow—A-Silikon—Vinyl Polysiloxan, Kulzer GmbH, Hanau, Germany, Fig. 1). This adjustment, which is similar to an impression, was made to soften the surface and ensure that the sensor was placed in a consistent position for each measurement and calibration process, thereby, improving the reproducibility of the measurements [42].

Patients were asked to bite three times with maximum force for a duration of 3 s. Pauses of at least 30 s were scheduled to avoid muscle fatigue. The highest score obtained was used for analyzing the maximum bite force. For the endurance test, patients were told to hold the adduction at 60% of the previously determined maximum bite force for as long as possible. The recording function of the I‑Scan software was used to open a real-time window where the patient could see the color pattern of their occlusal profile as visual feedback to help them maintain the defined force level. When the bite force dropped below 30% of the previously determined maximum bite force level, the time was stopped and used for further analysis.

**Fig. 2:**
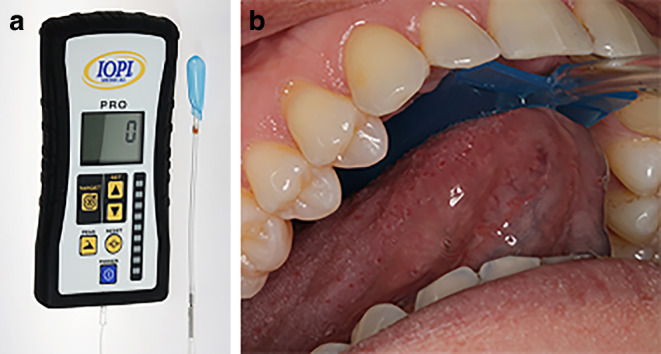
Measurement of maximum tongue pressure and tongue pressure endurance. Iowa Oral Performance Instrument Pro with indicator lights (vertical row of rectangular lights) on the liquid crystal display (LCD) for visual feedback during endurance measurement (**a**). The single air-filled bulb tongue array placed on the tongue blade 10 mm posterior of the tongue tip and 10 mm anterior to the circumvallata papilla (**b**) Messung von maximalem Zungendruck und maximaler Zungendruckausdauer. IOPI (Iowa Oral Performance Instrument) Pro mit Kontrollleuchten (vertikale Reihe von rechteckigen Lämpchen) auf dem LCD („liquid crystal display“)-Display zur visuellen Feedbackanzeige während der Ausdauermessung (**a**). Die mit Luft gefüllte Zungenblase wird 10 mm hinter der Zungenspitze und 10 mm vor der Papilla circumvallata platziert (**b**)

Maximum tongue pressure and tongue pressure endurance were measured using a handheld device (IOPI Medical LLC, Woodinville, WA, USA: Iowa Oral Performance Instrument, Fig. 2a). A single air-filled bulb tongue array was placed on the tongue blade 10 mm posterior of the tongue tip and 10 mm anterior to the circumvallata papilla (Fig. 2b; [43]). For maximum tongue pressure, patients were told to press their tongue against the air-filled bulb three times with maximum force and hold the pressure for 3–4 s, with a 30 s pause between each repetition. The highest value was used for analysis. For the endurance test, patients were asked to hold the pressure at 60% of the previously determined maximum value as long as possible. Indicator lights on the liquid crystal display (LCD) of the IOPI device served as visual feedback to make it easier for the patient to maintain the specified value (Fig. 2a). The time as outcome value for endurance was recorded equally as before: dropping from 60 to 30% stopped the clock. Active mouth opening was measured by a ruler at the mesioincisal angle of the upper and lower front teeth.

### Orthodontic examination

An orthodontic evaluation was performed clinically and with plaster casts. Orthodontic evaluation of the casts recording number of teeth, overbite and overjet, sagittal molar relationship and posterior crossbite was performed twice: a dentist and an orthodontist assessed all values separately, differences were then discussed and a consensus reading was obtained. The evaluation included an assessment of sagittal malocclusion, classifying the molar relationship according to Angle: class I, the mesiobuccal cusp of the first upper molar occludes in the buccal groove of the first lower molar; class II, the first lower molar is distally positioned relative to the first upper molar; and class III, the first lower molar is mesially positioned relative to the first upper molar. The original molar relationship was reconstructed in the case of extraction of premolars in the upper or lower jaw and in case of pronounced tooth migration. The vertical relationship of the anterior teeth was analyzed recording overbite, measured in millimeters as the vertical overlap of the incisal edges or space between the incisal edges. Overjet, the horizontal distance between the edge of the upper central incisor and the labial surface of the lower central incisor, was also measured in millimeters. Posterior crossbite was evaluated assessing the transversal relationship of the upper and lower premolars and molars. A crossbite was identified when the tips of the buccal cusps of one or more upper molars or premolars occluded within the central fossae of the lower molars or premolars. Conversely, the absence of a crossbite was defined as a condition where the tips of the buccal cusps of the lower premolars/molars were in contact with the central fossae of the opposing upper premolars/molars. A distinction was made between unilateral and bilateral posterior crossbites.

### Established motor scores

At both sites, subjects were prospectively assessed using the established motor scores (HFMSE, RULM, ALSFRS‑R and 6 MWT). The scores were rated by specially trained physiotherapists. Intra- and interrater reliability of the scores were described as excellent in various studies [31, 44–47]. All assessments were part of the routine procedures during patients’ visits for nusinersen injection or part of the natural history recording. The HFMSE was based on a 33-item scale covering activities of daily living, with each item being rated on a 3-point scale ranging from 0 to 2, resulting in a maximum total score of 66 [48]. The RULM, as a 19-item scale, was used to assess upper limb function. One item was scored as 0 or 1 points (can/cannot be realized), while all other items were scored on a 3-point scale (ranging from 0 to 2). The maximum total score was 37 [49]. The ALSFRS‑R, as a 12-item scale, addressed four domains (bulbar, upper limb, lower limb, respiratory). Each item was scored from 0–4 with a maximum total score of 48 [25]. For all three scores, a higher total corresponded to better motor function. The 6 MWT measured the distance (in meters) walked as quickly as possible over a 25-meter course in 6 min [47].

The BODS as an additional clinical outcome scale was administered by speech-language pathologists trained to assess dysphagia in patients with neuromuscular diseases. Patients’ ability to swallow saliva was rated in a first score BODS‑1 and their ability to consume liquids and foods in a second score BODS‑2. Both parts were scored from 1 (normal) to 8 (most severe) to indicate the patient’s degree of dysphagia [26, 27].

### Statistical analysis

Patient-specific oral function data from two visits were available. For each outcome, the mean value across the first and second measurement was used for analysis.

Possible floor and ceiling effects of the different outcomes were assessed via visual inspection using scatter plots with linear trend lines. For orientation, we considered a commonly used threshold for a ceiling/floor effect of 15% of the patients scoring at the lower/upper bound of the scale [21].

Differences in oral function scores based on patients’ orthodontic findings were analyzed using ordinary least squares (OLS) multiple regression. Unadjusted estimates were calculated to examine the bivariate associations between oral function scores and overbite, overjet, molar relationship (class I/III vs. class II), the presence of a posterior crossbite (no/yes), and history of orthodontic treatment (no/yes). Adjusted estimates were further analyzed by controlling for potential confounding factors, including ethnicity (Caucasian/Asian) and the number of teeth present. These models were designed to evaluate the independent effects of SMA-typical orthodontic findings on oral function while accounting for demographic and anatomical differences. Statistical significance was assumed at *p*-values < 0.05. All statistical analyses were conducted using SPSS (version 28.0.1.0, IBM, Armonk, NY, USA).

## Results

### Floor and ceiling effects

Compared with the oral function tests, a floor effect of the HFMSE score was found in the weakest sitters: 16 patients (38.1%) scored at a HFMSE score between 0 and 4, thereof 9 patients diagnosed with SMA type 2 and 7 patients with SMA type 3. Indication of a floor effect could also be found in the distribution of attained RULM-values. Nine of the 43 patients scored at a RULM score between 0 and 8, which corresponds to 20.9% of the sample. These 9 patients were also the weakest sitters and part of the group described above. Regarding the RULM score in comparison with the oral function tests, a ceiling effect was detected. In all, 13 patients (30.2%) scored with RULM values of 36 or 37 indicating low variability in less impaired patients with SMA type 3.

**Fig. 3:**
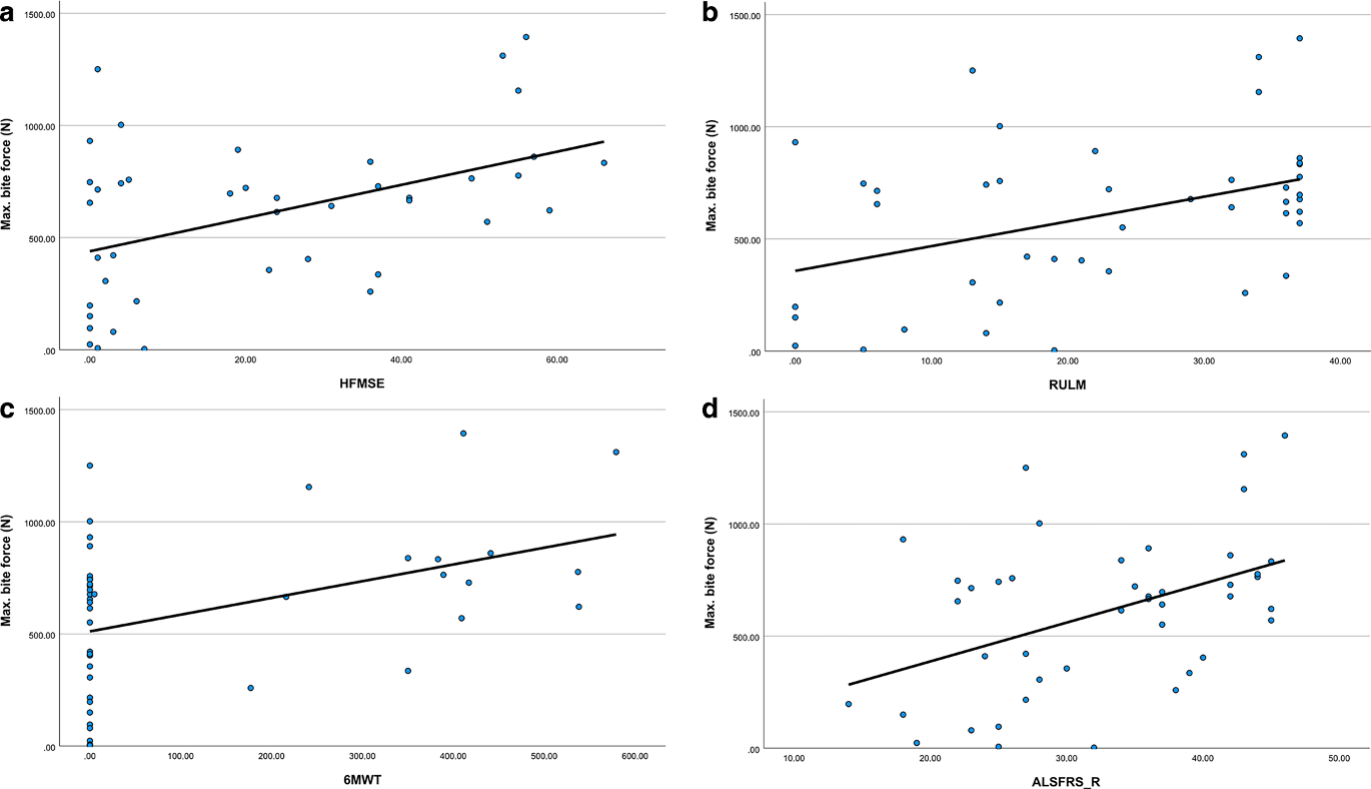
Linear associations between maximum bite force and established motor scores: **a** Hammersmith Functional Motor Scale Expanded (HFMSE), **b** Revised Upper Limb Module (RULM), **c** 6-Minute Walk Test (6 MWT), **d** Revised Amyotrophic Lateral Sclerosis Functional Rating Scale (ALSFRS-R) Lineare Assoziationen zwischen maximaler Kaukraft und etablierten motorischen Scores: **a** Hammersmith Functional Motor Scale Expanded (HFMSE), **b** Revised Upper Limb Module (RULM), **c** 6-Minuten-Gehtest (6 MWT), **d** Revised Amyotrophic Lateral Sclerosis Functional Rating Scale (ALSFRS-R)

**Fig. 4:**
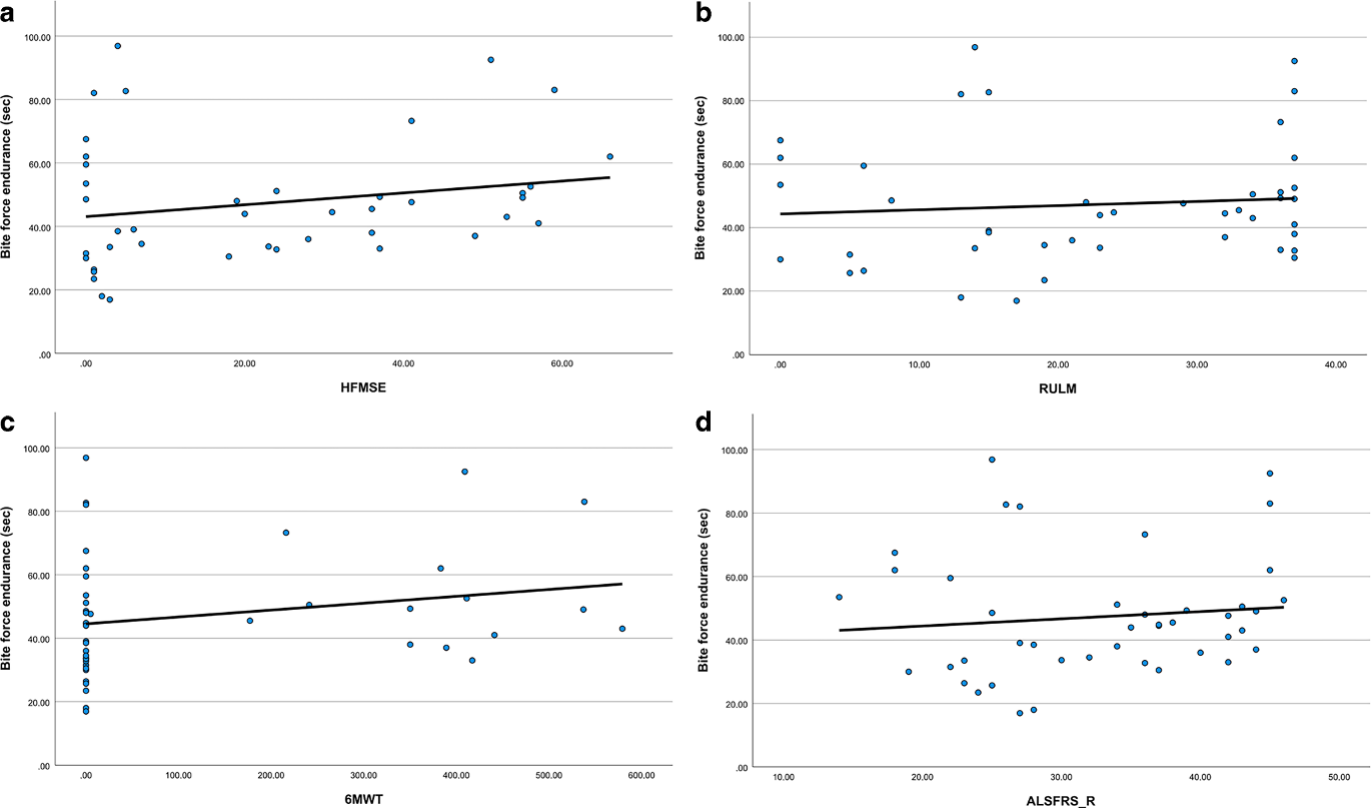
Linear associations between bite force endurance and established motor scores: **a** Hammersmith Functional Motor Scale Expanded (HFMSE), **b** Revised Upper Limb Module (RULM), **c** 6-Minute Walk Test (6 MWT), **d** Revised Amyotrophic Lateral Sclerosis Functional Rating Scale (ALSFRS-R) Lineare Assoziationen zwischen Kaukraftausdauer und etablierten motorischen Scores: **a** Hammersmith Functional Motor Scale Expanded (HFMSE), **b** Revised Upper Limb Module (RULM), **c** 6-Minuten-Gehtest (6 MWT), **d** Revised Amyotrophic Lateral Sclerosis Functional Rating Scale (ALSFRS-R)

**Fig. 5:**
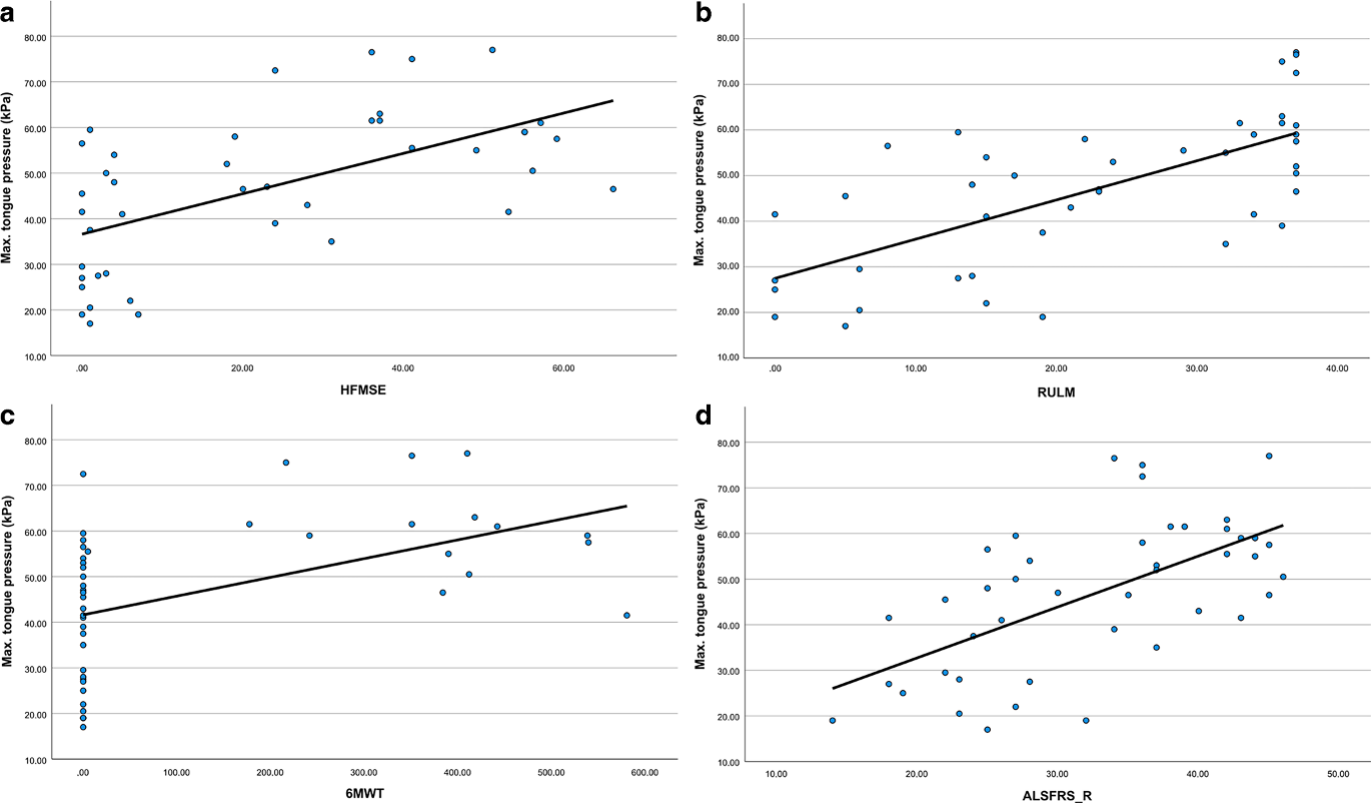
Linear associations between maximum tongue pressure and established motor scores: **a** Hammersmith Functional Motor Scale Expanded (HFMSE), **b** Revised Upper Limb Module (RULM), **c** 6-Minute Walk Test (6 MWT), **d** Revised Amyotrophic Lateral Sclerosis Functional Rating Scale (ALSFRS-R) Lineare Assoziationen zwischen maximalem Zungendruck und etablierten motorischen Scores: **a** Hammersmith Functional Motor Scale Expanded (HFMSE), **b** Revised Upper Limb Module (RULM), **c** 6-Minuten-Gehtest (6 MWT), **d** Revised Amyotrophic Lateral Sclerosis Functional Rating Scale (ALSFRS-R)

A total of 28 (65.1%) patients in our sample were nonambulatory (12 SMA type 2 and 16 SMA type 3). Although the 6 MWT score could not be applied in this subgroup, the lack of differentiation (maximal floor effect) in nonambulatory patients is shown in Figs. 1c, 2c, 3c, 4c and 5c, describing at the same time the variability of the observed oral function values.

No indication of floor or ceiling effects could be observed concerning the association between the oral function measures and the ALSFRS‑R score. The result of the BODS score showed a major lack of discrimination of the functional (swallowing) status in the patients, which affected the whole range of the scale: 36 of the 43 patients (83.7%) scored at a value of 2 (not shown).

The association between maximum bite force and the HFMSE score (Fig. 3a) indicated little variation in HFMSE values in the range of HFMSE scores less than 5. In these weakest sitters, maximum bite force was measured between 2.7 and 1250.5 N compared to bite force levels in the literature measured over the total dental arch of up to 905–1181 N in healthy subjects [50]. Three of the 4 patients scoring at 0 in the RULM test had very low bite force values ranging between 23.5 and 197.0 N. The fourth patient with SMA type 2 showed a bite force level of 930.9 N, which was within the range of values found in healthy subjects, more precisely, even above the mean value of 595.2 N found in healthy volunteers (author’s data, not shown). At the upper end of the RULM scale, Fig. 3b shows the limited discrimination of the RULM score at values above 35. Maximum bite force values ranging between 335.4 and 1394.1 N showed no ceiling effect (Fig. 3b).

The superior discriminatory power of bite force measurement in strongly impaired patients with SMA became particularly apparent in the subgroup of nonambulatory patients (excluded for the 6 MWT test, but shown in Fig. 3c with 0 m walking distance) where maximum bite force values varied between 2.7 and 1250.5 N.

Similar patterns were found in the association between bite force endurance and the established motor scores (Fig. 4). In the subgroup of the weakest sitters (HFMSE score < 5), bite force endurance varied between 17.0 and 96.9 s (Fig. 4a). The 4 patients who scored 0 on the RULM test had bite force endurance ranging from 30.0 to 67.5 s, indicating no floor effect in bite force endurance (Fig. 4b). At the upper end of the RULM scale, Fig. 4b again shows the limited discrimination of the RULM score at values above 35. Bite force endurance values ranging between 30.5 and 92.5 s showed no ceiling effect (Fig. 4b). In the subgroup of nonambulatory patients, the full range of 17.0 to 96.9 s bite force endurance was represented (Fig. 4c).

Patients with a HFMSE score < 5 achieved maximum tongue pressures ranging from 19.0 to 59.5 kPa (Fig. 5a). The 4 patients with a RULM score = 0 were in the lower range of maximum tongue pressures with values between 19.0 and 41.5 kPa (Fig. 5b). At the upper end of the RULM scale above 35, maximum tongue pressures ranged from 39.0 to 77.0 kPa and showed no ceiling effect (Fig. 5b). In the subgroup of nonambulatory patients, maximum tongue pressures ranged from 17.0 to 72.5 kPa (Fig. 5c).

**Fig. 6:**
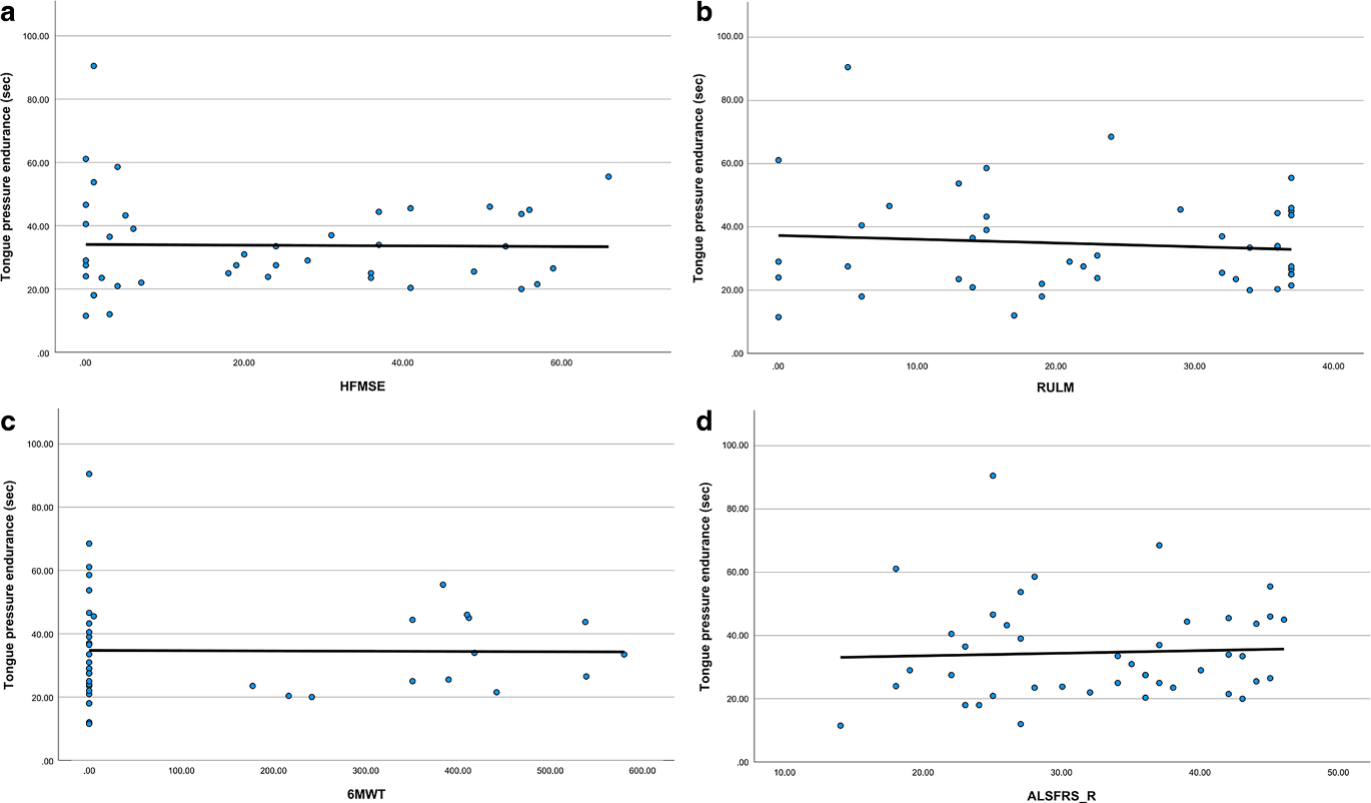
Linear associations between tongue pressure endurance and established motor scores: **a** Hammersmith Functional Motor Scale Expanded (HFMSE), **b** Revised Upper Limb Module (RULM), **c** 6-Minute Walk Test (6 MWT), **d** Revised Amyotrophic Lateral Sclerosis Functional Rating Scale (ALSFRS-R) Lineare Assoziationen zwischen Zungendruckausdauer und etablierten motorischen Scores: **a** Hammersmith Functional Motor Scale Expanded (HFMSE), **b** Revised Upper Limb Module (RULM), **c** 6-Minuten-Gehtest (6 MWT), **d** Revised Amyotrophic Lateral Sclerosis Functional Rating Scale (ALSFRS-R)

Tongue pressure endurance measured in patients with a HFMSE score < 5 as well as in the subgroup of nonambulatory patients showed a wide range from 11.5 to 90.5 s (Fig. 6a and c). The endurance values of the four patients with a RULM score = 0 ranged from 11.5 to 61.1 s and those of the 13 patients with a RULM score > 35 ranged from 20.35 to 55.5 s (Fig. 6b).

**Fig. 7:**
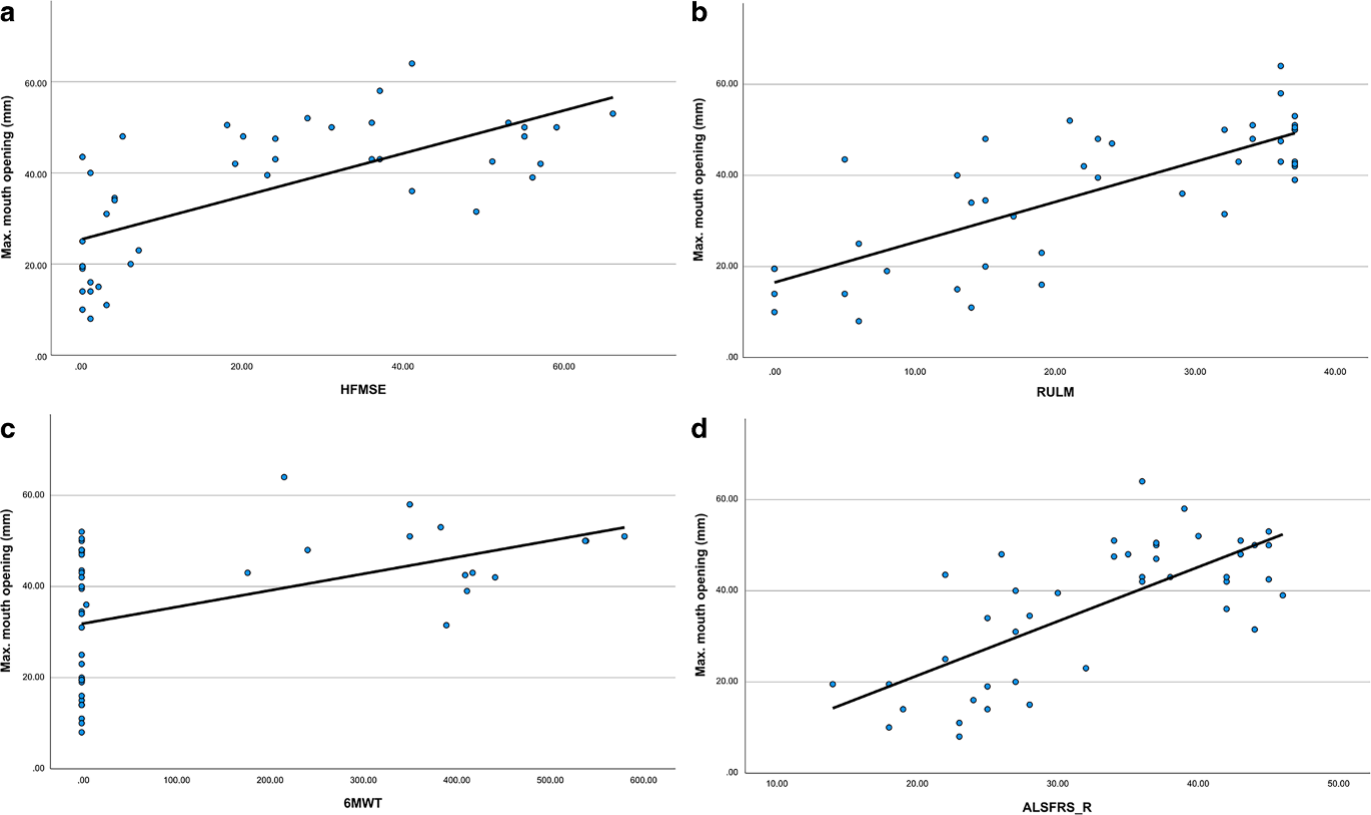
Linear associations between maximum mouth opening and established motor scores: **a** Hammersmith Functional Motor Scale Expanded (HFMSE), **b** Revised Upper Limb Module (RULM), **c** 6-Minute Walk Test (6 MWT), **d** Revised Amyotrophic Lateral Sclerosis Functional Rating Scale (ALSFRS-R) Lineare Assoziationen zwischen maximaler Mundöffnung und etablierten motorischen Scores: **a** Hammersmith Functional Motor Scale Expanded (HFMSE), **b** Revised Upper Limb Module (RULM), **c** 6-Minuten-Gehtest (6 MWT), **d** Revised Amyotrophic Lateral Sclerosis Functional Rating Scale (ALSFRS-R)

Maximum mouth opening varied between 8.0 and 43.5 mm in the patients demonstrating a HFMSE score < 5 (Fig. 7a). Ten of the 16 patients had a mouth opening smaller than 20.0 mm, which is a major restriction compared to the mean of maximum mouth opening in healthy subjects (specified in the literature with 55 mm [33]). The 4 patients with a RULM score = 0 showed a maximum mouth opening between 10.0 and 19.5 mm (Fig. 7b). At the upper end of the RULM scale above 35, maximum mouth opening ranged between 39.0 mm and the above-average value of 64.0 mm (Fig. 7b). The subgroup of nonambulatory patients scored between 8.0 and 52.0 mm (Fig. 7c).

### Orthodontic results

The patients’ mean number of teeth was 27.4 ranging from 23 to 32. Moderate to severe malocclusions, typical of SMA, were present in the sample: 22 patients (51.2%) had a class II molar relationship with a mean overjet of 5.7 mm ranging from 2 to 15 mm. Eleven patients (25.6%) had an overbite of 0 mm or an open bite (mean overbite of −1.5 mm ranging from −5 mm to 0 mm). Twenty patients had a posterior crossbite (46.5%), thereof 12 with a unilateral and 8 with a bilateral crossbite (Table 2).

**Table 2:** Orthodontic findings in the spinal muscular atrophy (SMA) sample Kieferorthopädische Befunde der SMA(spinale Muskelatrophie)-Gruppe

		*N*	(%)	Mean	SD	Median	Min	Max
Number of teeth		43	–	27.4	2.2	28	23	32
Overbite		43		1.4	2.1	2	−5	5
Overjet		43		3.9	3.6	3	−3	15
Orthodontic treatment	No	21	(51.2%)	–				
	Yes	20	(48.8%)					
Molar relationship	Class I	18	(41.9%)					
	Class II	22	(51.2%)					
	Class III	3	(7.0%)					
Posterior crossbite	None	23	(53.5%)					
	Unilateral	12	(27.9%)					
	Bilateral	8	(18.6%)					

*SD* standard deviation; *N* sample size; *Min* minimum; *Max* maximum

Analysis revealed statistically significant associations between different malocclusions and reduced oral function measures in SMA patients. Lower values of maximum bite force, maximum tongue pressure, maximum mouth opening, and bite force endurance were observed in patients with a larger overjet (*p*-values < 0.05, except for the adjusted estimates for maximum bite force and bite force endurance, Table 3). Class II molar relationship (as a second measure of a sagittal malocclusion) was negatively associated with maximum bite force (*p* = 0.006 unadjusted, *p* = 0.015 adjusted) and maximum tongue pressure (*p* < 0.001 for both unadjusted and adjusted calculations). Similarly, a posterior crossbite correlated with reduced maximum tongue pressure (*p* = 0.021 unadjusted calculation, *p* = 0.015 adjusted calculation) and maximum bite force after adjusting (*p* = 0.043). Almost all associations remained robust after adjusting for differences in the number of teeth and ethnicity (Table 3). In contrast, no significant associations were identified between oral function measures and a history of orthodontic therapy (*p* > 0.05, data not shown). Detailed results are provided in Table 3.

**Table 3:** Ordinary least squares (OLS) models regressing oral function measures on orthodontic findings OLS („ordinary least squares“)-Modelle zur Regression von oralen Funktionswerten auf kieferorthopädische Befunde

	Unadjusted				Adjusted			
	Coef	Lower CI	Upper CI	*p*-value	Coef	Lower CI	Upper CI	*p*-value
*Maximum bite force*								
Overbite	3.154	−50.089	56.397	0.905	9.789	−41.404	60.981	0.701
Overjet	−39.943	−68.409	−11.477	0.007^*^	−30.823	−62.572	0.925	0.057
Molar relationship^1^	−290.726	−492.581	−88.870	0.006^*^	−249.425	−446.525	−52.326	0.015^*^
Posterior crossbite^2^	−194.203	−407.570	19.165	0.073	−205.908	−404.864	−6.951	0.043^*^
*Bite force endurance*								
Overbite	−0.504	−3.384	2.377	0.725	−0.130	−3.018	2.758	0.928
Overjet	−1.827	−3.414	−0.240	0.025*	−1.782	−3.564	0.001	0.050
Molar relationship^1^	−5.042	−17.226	7.143	0.408	−4.285	−16.244	7.673	0.473
Posterior crossbite^2^	−5.053	−17.237	7.132	0.407	−5.081	−16.817	6.656	0.386
*Maximum tongue pressure*								
Overbite	−0.006	−2.392	2.381	0.996	0.184	−2.214	2.582	0.877
Overjet	−2.380	−3.550	−1.210	< 0.001^*^	−2.409	−3.747	−1.070	<0.001^*^
Molar relationship^1^	−11.109	−20.505	−1.713	0.022^*^	−9.822	−19.352	−0.292	0.044^*^
Posterior crossbite^2^	−11.180	−20.567	−1.792	0.021^*^	−11.558	−20.707	−2.409	0.015^*^
*Tongue pressure endurance*								
Overbite	−0.374	−2.818	2.071	0.759	−0.259	−2.771	2.253	0.836
Overjet	0.489	−0.939	1.918	0.493	0.659	−0.961	2.279	0.415
Molar relationship^1^	2.483	−7.672	12.639	0.624	2.441	−7.953	12.835	0.637
Posterior crossbite^2^	2.191	−7.972	12.353	0.665	2.319	−7.915	12.554	0.649
*Maximum mouth opening*								
Overbite	0.493	−1.734	2.720	0.657	0.649	−1.649	2.948	0.571
Overjet	−1.945	−3.094	−0.795	0.001^*^	−2.171	−3.488	−0.855	0.002^*^
Molar relationship^1^	−7.382	−16.554	1.790	0.112	−6.891	−16.323	2.540	0.147
Posterior crossbite^2^	−6.316	−15.569	2.937	0.175	−6.427	−15.740	2.886	0.170

*Lower/Upper CI* Lower/Upper 95% confidence interval

^1^Class II vs class I/III

^2^Uni-/bilateral vs. none

^*^p-value < 0.05; adjusted estimates account for differences in the number of teeth and ethnicity

## Discussion

In this study, we demonstrated that oral function tests can be performed in a subgroup of severely affected SMA patients, where established motor scores reach their limits.

The floor effect of the HFMSE test found in our data is in line with previous work [31], describing the same cut-off values of a HFMSE score < 5 when compared with muscle strength measures [28]. A ceiling effect of a RULM score > 35 as observed in our data has previously been found in up to a third of ambulatory SMA type 3 patients without upper limb weakness [29, 31]. Our results show that in contrast, oral function tests bear very little risk of floor or ceiling effects in the subgroup of adult SMA type 2 and 3 patients. They are able to detect small differences in oral function providing data over a continuous range. Lower oral function values were associated with the presence of an enlarged overjet, a class II molar relationship and a posterior crossbite. These malocclusions are typically the result of skeletal dysgnathia in patients with neuromuscular disorders and are already known in SMA [51]. They should be given special consideration in the context of neuromuscular assessment.

As all motor scales have their strengths and limitations, selecting and combining the best outcome measures in (severely affected) adult SMA patients is challenging, but of enormous importance in order to assess the efficacy of treatment options. Beyond that, the additional information on oral function should be considered in every patient evaluation. Whether used in combination or alone, several reasons favor oral function tests over other established motor scores. First, oral function is affected at a later stage and less severely than axial or proximal muscle groups, which explains why no values of 0 N or 0 kPa were measured. Second, continuous scales are used, so that even small forces of, for example 2.7 N, can be measured, avoiding the bunching of many patients at the same score. Third, the straightforward collection of both absolute maximum measures and endurance data with the same devices is of great diagnostic potential, as endurance tests reflect an important additional dimension of physical impairment in SMA beside muscle strength. Fourth, distinctive characteristics of neurodegenerative diseases or especially of SMA may lead to methodologically specific inaccuracies measuring severely restricted general motor function. Patients with SMA usually have a normal coordinative function. However, in cases of severely impaired muscle strength, motor coordination in certain motor score tasks may be hampered by this alone, may prevent the achievement of a minimum score and therefore enhance a floor effect [52]. Our simple muscle strength and endurance tests reduce this risk due to their simple series of minimal movements during isometric contraction and the guidance/orientation provided by the measuring instrument itself. In the same vein, spinal fusion, contractures, scoliosis or other comorbidities can influence motor function scores as they interfere with some tasks, e.g. rolling [31, 53].

Our clinical observations indicate that ambulatory patients rarely report dysphagia as a sign of bulbar involvement. Likewise, previous findings indicated that ambulant SMA patients achieved the same masticatory performance as healthy probands, despite their abnormalities in muscle structure [7]. Our analyses corroborate these findings and show unrestricted results of oral function tests in some exceptional cases. Outliers in the SMA group showed even higher values than those expected for healthy subjects. There is currently no definitive explanation for this.

Factors such as age, gender, ethnicity or malocclusion can affect maximum bite force and other oral function values [16, 54–56]. Clearly, the most relevant factor seems to be the number of tooth contacts [16, 57], which can vary considerably in SMA patients. Reduced tooth contacts may first be due to tooth loss resulting from poor oral hygiene. With an only moderately reduced number of teeth (range 23–32), tooth loss was no decisive factor in our sample and was proactively controlled for in the OLS models. Second, reduced tooth contacts may depend on the malocclusion, which was part of our research question. In this vein, craniofacial anomalies and malocclusions were found more frequently in the SMA sample than in the average population. The prevalence of anterior open bite is reported to be between 1.5 and 11% depending on age and ethnicity [58] compared to 25.6% of the patients having an overbite between 0 and −5 mm in our SMA sample. The reported prevalence of class II malocclusions varies greatly depending on ethnicity, ranging from 18.5% in Colombia [59] to 48.8% in Sweden [60]. Relative to these values, the SMA sample examined in this study showed a higher proportion of a class II molar relationship (51.2%). As 85.7% of our sample were Caucasian, a bias in the malocclusion frequencies is rather unlikely; OLS models were adjusted for ethnicity.

A class II molar relationship, an enlarged overbite, a posterior crossbite and an open bite are known to be associated with a reduced bite force and tongue pressure in healthy individuals [15, 16, 61]. Our data show statistically significant associations between lower oral function values and the presence of an enlarged overjet, a class II molar relationship, and a posterior crossbite. They thus confirm the results from previous studies, irrespective of the patient’s underlying disease [15, 16, 61, 62].

On the one hand, a clear distinction between anatomical oral dysfunction due to malocclusion or a reduced number of teeth and neuromuscular oral dysfunction is not possible without additional, more complex measurement methods. This can lead to inaccuracies in the interpretation of oral function data. On the other hand, altered craniofacial anatomy and tooth loss in SMA is causally related to neuromuscular dysfunction and thus reflect the severity of the neuromuscular disease [12, 13]. It is therefore unclear whether a strict separation is necessary.

The additional recording of a surface electromyography (sEMG) could have provided important information to differentiate between the anatomical–mechanical disadvantage of the facial skull structure and the neuromuscular deficit of the patients. The relationship between bite force generation and the level of muscular effort (EMG/force slopes) could have further strengthened our results of the oral function tests and ruled out bias due to patient’s motivation or reaction to visual feedback [63]. Notably, however, Florence et al. concluded that for maximum bite force measurements in patients with myasthenia gravis, sEMG recordings and bite force measurements yielded comparable information, suggesting that either method can effectively capture relevant data [42].

A few methodological limitations in the measurement of oral function may apply due to the typical dentofacial anatomy of SMA patients. In addition to the partly pronounced skeletal malocclusions, patients with a class II molar relationship frequently presented with protrusive, flared upper incisors. This anterior tooth position could have affected the measurement of overjet, open bite and maximum mouth opening. The typically narrow and high palate made it difficult to place the IOPI bulb, as it already has a tendency to slip off the tongue. In addition, the high palate may have increased difficulties for the patient to exert full force with the tongue, which would once again result in lower force values measured for anatomical reasons.

For endurance, we chose a target value of 60% of the previously determined maximal force. This protocol is consistent with previous approaches [34], but can be questioned for two reasons. First, methodologically inaccurate maximal values may result in biased endurance measurements. Second, interindividual differences may have been more pronounced if patients had been asked to generate an absolute predefined force level.

As another limitation of this study was the fact that the assessment of malocclusion was based solely on clinical findings and model analysis. As the patients were not undergoing orthodontic treatment, lateral cephalograms were not available to differentiate between skeletal and dental malocclusions, neither in the sagittal nor in the vertical dimension. Lateral and axial cephalometric data, such as those available in a comparable study on Duchenne patients [38], would be of great value to make reliable statements about the skeletal component of the present malocclusions.

While the relatively small sample size may be seen as another limitation, it is comparable to existing observational studies and must be seen in light of the low incidence of the disease. No sample size analysis was performed due to the limited number of eligible patients to be enrolled at the two participating study sites. Still, the realized sample included a heterogeneity of patients, differing in SMA type and treatment status, which allowed us to assess the applicability of oral function tests across a wide range of motor abilities, which is important to identify floor or ceiling effects. To unlock the full diagnostic potential of oral function tests, future studies with larger sample sizes should go beyond the cross-sectional design chosen here to track changes over time.

## Conclusion

This study suggests oral function tests that offer a suitable method of measuring oral function in SMA without the risk of a floor or ceiling effect. In severely affected SMA patients oral function tests may help to assess progressive degeneration or therapeutic effects by closing a diagnostic gap often described in prior studies. When established motor scores are no longer able to discriminate between patients or to reveal changes within one patient due to a floor effect, oral function tests still provide data over a continuous range detecting even small differences.

In combination with the causal therapy now available for SMA since birth, early orthodontic therapy should be considered for affected children. This could reduce the severity of skeletal dysgnathia and oral dysfunction and subsequently prevent a decrease in bite force and tongue pressure for anatomical reasons alone. If skeletal changes could be prevented in this way, monitoring of neuromuscular alterations of oral function would be less biased.

## Data Availability

The datasets used and/or analyzed during the current study are available from the corresponding author on reasonable request.
